# Performance evaluation of a CD34 expression assay by digital PCR in bone marrow harvest from healthy donors

**DOI:** 10.1111/trf.70267

**Published:** 2026-06-01

**Authors:** Miguel Waterhouse, Anja Kaufmann, Martina David, Sabine Enger, Melanie Sieder, Darina Siegmund, Milena Pantic, Justyna Rawluk, Robert Zeiser, Claudia Wehr, Ralph Wäsch, Justus Duyster, Jürgen Finke, Chrissoula Kiote‐Schmidt

**Affiliations:** ^1^ Department of Hematology, Oncology and Stem Cell Transplantation Freiburg University Medical Center Freiburg Germany

**Keywords:** bone marrow, CD34 expression, healthy donors

## Abstract

**Background:**

The aim of this study was to investigate a CD34 expression assay as an additional tool for the total nucleated cell (TNC) count assessment in bone marrow harvest (BMH) obtained from healthy donors.

**Study Design and Methods:**

Samples obtained from bone marrow donors (*n* = 71) were analyzed for CD34 expression using a digital PCR assay. The assay was extensively validated and the obtained data was correlated with standard BMH assessment methods such as TNC counts performed using a hematology analyzer and CD34 cell count measured with flow cytometry.

**Results:**

The digital PCR CD34 assay showed a strong correlation with the TNC count (Spearman's *r*s 0.87, 95% CI: 0.78–0.92, *p* < .001) and also with the CD34 cell count assessed by flow cytometry (Spearman's *r*s 0.91, 95% CI: 0.84–0.94, *p* < .001). To analyze the diagnostic performance of the assay, the true‐positive rate (sensitivity) and the false‐positive rate (1 − specificity) were plotted in a receiver‐operating characteristic (ROC) space.

The ROC curve showed an area under the curve (AUC) of 0.96 (95% confidence interval 0.91–1.01, *p* < .005). The CD34 NCN per kilogram of recipient body weight threshold of 0.54 resulted in a sensitivity of 0.91, a specificity of 0.96, a positive and negative predictive value of 0.97 and 0.91, respectively.

**Conclusions:**

The CD34 expression assay here described can be used as an additional tool for TNC count estimation after BMH.

**Abbreviations**
AUC: area under the curve
BMH: bone marrow harvest
CLSI: Clinical and Laboratory Standards Institute
CV: coefficient of variation
GvHD: graft‐versus‐host disease
HSCT: allogeneic stem cell transplantation
LoB: limit of blank
LoD: limit of detection
LoQ: limit of quantification
MU: measurement uncertainty
NCN: normalized copy number
ROC: receiver‐operating characteristic
TNC: total nucleated cell

## 1 INTRODUCTION

With the advent of granulocyte‐colony stimulating factor in the early 90s, mobilized peripheral blood (PB) has become the main source of hematopoietic stem cells for allogeneic stem cell transplantation (HSCT).^1^ Despite the increasing number of mobilized PB for HSCT, marrow still remains a relevant graft source for diseases such as aplastic anemia and for pediatric transplantation where graft‐versus‐host disease (GvHD) represents a clinical concern.^2^, ^3^

Bone marrow harvest (BMH) quality assessment includes, among other parameters, the total number of nucleated cells (TNC) collected during and after the procedure.^4^ The TNC count is a key factor for successful engraftment and as previously suggested TNC counts requirement for HSCT lies between 2 and 4 × 10^8^ TNC per kilogram of recipient body weight.^5^, ^6^, ^7^ The TNC composition includes different cell types, like lymphocytes, monocytes, neutrophils, and non‐hematopoietic cells, such as mesenchymal stem cells.^8^ From this complex mixture of cells, only a minor fraction of them, represented by the stem/progenitor cells, have engraftment and long‐term repopulation capabilities.^9^, ^10^

TNC counts are typically performed using hematology analyzers. However, hematology analyzers employ different technologies to assess cell counts such as impedance and optical methods like light scattering and flow cytometry. This technological diversity results in TNC count discrepancies between the BMH collection facilities and the HSCT centers where they usually confirm the TNC count generated at the collection timepoint.^11^, ^12^

Additional inconsistencies in the TNC counts may arise from transportation delays, mechanical stress, microclots formation during transport, hyperlipidemia, and platelet aggregates among others.^13^ Moreover, it is unclear to what degree these factors may affect the different cell subpopulations contained in the collected marrow.

These disparities are difficult to resolve because the assessment of the TNC count at the collection timepoint is not representative of a similar count performed with a retained BMH sample when the marrow infusion is carried out afterwards, even using the same hematology analyzer due to the extemporaneous nature of the sample.

Therefore, an additional mean of the TNC count that reflects the cell count at the time of collection in particular those cells with long‐term repopulation potential is highly desirable. CD34 expressed on the hematopoietic stem cells is widely accepted as a marker for stem cells with regenerative capacity and has been used to predict engraftment following HSCT.^9^, ^10^

The aim of our study was to investigate a CD34 expression assay as an additional strategy for the BMH TNC count assessment.

## 2 MATERIALS AND METHODS

### 2.1 Donor's characteristics and samples

A total of 71 BMH procedures from healthy donors were performed in our collection center. The BMH product was obtained using a closed collection system (BioAccess, Baltimore, USA). Before the BMH procedure, clinical and laboratory examination, including infectious markers, were performed in all donors according to institutional guidelines. The BMH procedure was done under general anesthesia and after thorough skin disinfection, a bone marrow harvest needle (MDL, Delebio, Italy) was used over the superior iliac crest to collect the marrow. After the BMH procedure, the marrow contained in the primary collection bag was filtered to remove bone fragments and blood clots. This filtering system was connected with the final product bag, from where samples were obtained for microbiological controls and cell counts (Appendix S1). The remains of these samples (3–5 mL) were used to establish the assay here described.

This study was approved by the Ethics Committee of the Albert Ludwigs University, Freiburg, Germany (Nr 22‐1490‐S1). Written informed consent was obtained from the donors in accordance with the Declaration of Helsinki.

### 2.2 TNC, CD34 and CD3 cell counting

TNC cell counts were performed with an automated hematology analyzer after the BMH procedure (Sysmex GmbH, Norderstedt, Germany), and the TNC was calculated by multiplying the cell number concentration for the total volume of the collection bag. The CD34 cell count was determined in the same sample with the CD34 stem cell enumeration kit containing also a CD45 antibody and 7‐ADD for cell vitality assessment (Becton Dickinson, Heidelberg, Germany). CD3 cell counts were measured separately according to an in‐house protocol. All cell measurements were performed on a FACS Lyric flow cytometer, and the data thereof was analyzed using the FACSuite software (version 1.5, Becton Dickinson, Heidelberg, Germany).

### 2.3 RNA isolation, RT‐PCR and CD34 expression

Total RNA was extracted from the BMH samples and from several cell lines with the QIASymphony RNA kit (Qiagen GmbH, Hilden, Germany) following the manufacturer's instructions. The extracted RNA was reverse transcribed using random hexamer primers with the Maxima First Strand cDNA synthesis kit (Thermo Fisher Scientific GmbH, Dreieich, Germany). A minimum of 1 μg of RNA template was mixed with 1 μL of 10× ds DNAse buffer and 1 μL of DNAse, in a final volume of 10 μL of RNase‐free water and was incubated for 10 min at 37°C. The samples were then chilled followed by the addition of 4 μL 5× reaction mix, 2 μL reverse transcriptase enzyme and 4 μL nuclease‐free water. Samples were incubated for 10 min at 25°C followed by 30 min at 50°C. The reaction was terminated by incubating the samples at 85°C for 5 min. The synthesized cDNA was stored at −80°C until further analysis.

The digital PCR reaction (dPCR) volume for the CD34 expression assay was 20 μL, composed of 10 μL of dPCR supermix for probes (Bio‐Rad Laboratories, Munich, Germany), 3 μL of cDNA and primer and probes concentration as already described.^14^ Human *ABL* gene was used as the reference gene. Each reaction mixture was partitioned into approximately 20,000 droplets using a droplet generator (Bio‐Rad Laboratories, Munich, Germany) and then cycled under the following conditions: 95°C for 10 min, followed by 40 cycles of 94°C for 30 s, 60°C for 1 min and one final cycle of 98°C for 10 min.

Cycled droplets were read in a QX200 droplet‐reader and the analysis of the dPCR data was performed using the QuantaSoft analysis software (Bio‐Rad Laboratories, Munich, Germany). The threshold between the positive and negative droplet clusters was manually set for each fluorochrome channel. Assay results were reported as the normalized copy number (NCN) of CD34 mRNA copies relative to *ABL* transcripts (NCN = CD34 copy number/ABL copy number × 100) and also as CD34 NCN per kilogram of recipient body weight. Additional assay optimization such as annealing temperature determined by the resolution factor is described in Appendix S1.

### 2.4 CD34 assay performance characteristics

The limit of blank (LoB), limit of detection (LoD), and limit of quantification (LoQ) were established as suggested by the Clinical and Laboratory Standards Institute (CLSI guideline EP 17 A2, EP 06)^15^ using the precision profile approach and also by the European guidelines for real‐time PCR.^16^ Briefly, a high CD34 expressing sample was serially diluted with cDNA obtained from the K562 cell line (which does not express CD34), resulting in five CD34 cDNA concentration levels. Each dilution from these artificial mixtures was analyzed in five replicates. The LoB was assessed by measuring false positive events from a series of 10 cDNA obtained from the K562 cell line. Additional cell lines (REH, THP‐1, NB‐4, OCI‐AML2) without CD34 expression were also tested. For the assay linearity and dynamic range study, intermediate and high cDNA CD34 concentrations were used covering the analytical measuring interval. Measurement uncertainty (MU) was assessed using two levels of CD34 expressing samples obtained from the serial dilutions mentioned. Each of these levels was tested in 20 replicates for intra and interassay imprecision (CLSI guideline EP29A).^17^

### 2.5 Statistical analysis

Common statistical parameters were calculated using Analyse‐it software version 5.51 (Analyse‐it, Leeds, United Kingdom) and the Abacus software version 3.0 (LABanalytics GmbH, Jena, Germany). Correlation coefficients were calculated by the Spearman rank correlation coefficient analysis. For the comparison of qualitative or quantitative variables without a normal distribution, the Mann–Whitney, Wilcoxon signed‐rank test, or linear regression tests were used. Quantitative variables were analyzed with the Student paired *t*‐test or Fisher's exact test in the case of small numbers. All tests were two‐sided, accepting *p* ≤ .05 as indicating a statistically significant difference. General performance of the CD34 expression assay was analyzed by plotting the true‐positive rate (sensitivity) and the false‐positive rate (1 − specificity) in a receiver‐operating characteristic (ROC) space.

## 3 RESULTS

### 3.1 Donor and BMH characteristics

Before and during the BMH procedure, no complications in the donors were detected both during the anesthesia and the marrow aspiration. After the BMH procedure, with the exception of controllable pain at the puncture site, no serious adverse effects in the donors were reported. Donors were fully recovered and discharged from the hospital after the BMH. Clinical and blood controls were recommended after hospital discharge by the attending physician. Donor and recipient characteristics are summarized in Table 1. Details of the analyzed samples obtained after the BMH can be found in Table 2.

**TABLE 1:** BM donor and recipient characteristics.

Total marrow donors	71
Donor sex	
Female (%)	27 (38)
Male (%)	44 (62)
Mean donor weight in kg (range)	
Female	67.1 (63–88)
Male	82.9 (62–120)
Mean recipient weight in kg (range)	42.4 (6–102)
Mean donor age (range)	29.2 (20–46)
Mean duration of BMH^a^ (range)	30.4 (15–87)

Abbreviation: BMH, bone marrow harvest.

^a^ Results are expressed in minutes.

**TABLE 2:** BMH samples characteristics.

	Mean value (range)
TNC (10^10^ cells)	1.43 (0.28–3.53)
TNC/mL (10^7^ cells)	1.49 (0.74–3.32)
TNC/kg^a^ (10^8^ cells)	4.75 (1.12–14)
CD34 (%)^b^	0.92 (0.38–2.06)
CD34/mL (10^5^ cells)	1.14 (0.045–3.68)
CD34 total (10^8^ cells)	1.5 (0.28–5.26)
CD34/kg (10^6^ cells)	4.47 (1.05–17.5)
CD34 vitality (%)	96.8 (92–98)
CD3 total (10^8^ cells)	15.8 (3.33–31.5)
CD3/mL (10^5^ cells)	13.1 (1.69–20.3)
CD3/kg (10^6^ cells)	47.8 (5.51–118)
BMH volume (mL)	1191.3 (338–1715)
BMH volume/kg donor	15.8 (3.35–23.9)

Abbreviations: BMH, bone marrow harvest; TNC, total nucleated cells.

^a^ kg of recipient body weight.

^b^ Expressed as % of CD45+ cells.

### 3.2 CD34 expression assay analytical characteristics

CD34 expression specificity was assessed using several cell lines which are known for the lack of CD34 expression (K562, REH, THP‐1, NB‐4, OCI‐AML2). In none of the tested cell lines were CD34‐positive droplets detected.

The LoB using 10 cDNA obtained from the K562 cell line, tested in duplicates, resulted in no positive droplets for the CD34 cDNA target.

The LoD for the CD34 expression, using serial CD34 cDNA dilutions resulted in a CD34 NCN of 0.048. To assess the LoQ, we followed the European guidelines for real‐time PCR methods. Following this recommendation, the LoQ is represented by the lowest NCN value in which the coefficient of variation (CV) is below 25%. Therefore, the LoQ of our assay was a CD34 NCN of 0,094 (Figure 1). All the tested samples obtained from the BMH were above the CD34 expression assay LoQ (mean CD34 NCN 23.1 range: 10.6–41.2). The mean donor CD34 NCN per kilogram of recipient body weight was 0.87 (range: 0.22–326).

**FIGURE 1:**
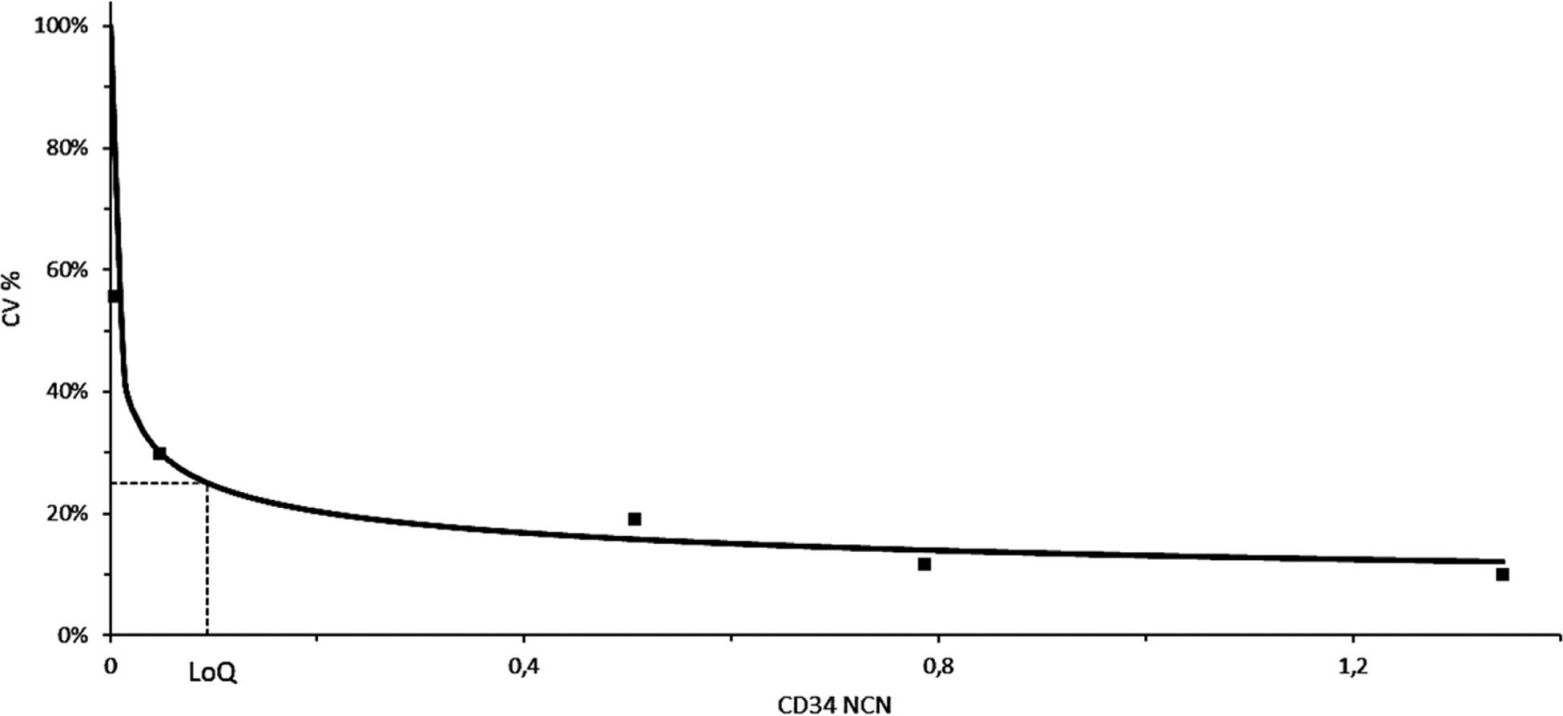
CD34 dPCR assay limit of detection and limit of quantification. A precision profile was generated using a sample with high CD34 expression, serially diluted in mRNA extracted from the K562 cell line. To establish the LoD and LoQ, an alternate three parameter variance function was applied. This statistical approach is recommended when the values are near zero. CV%, coefficient of variation (percentage).

The assay dynamic range was assessed by plotting the expected CD34 concentration (NCN range: 3–28) against the measured CD34 concentration. This measurement resulted in a line with a slope of 0.981 that intercepts at 0.143 (Figure 2).

**FIGURE 2:**
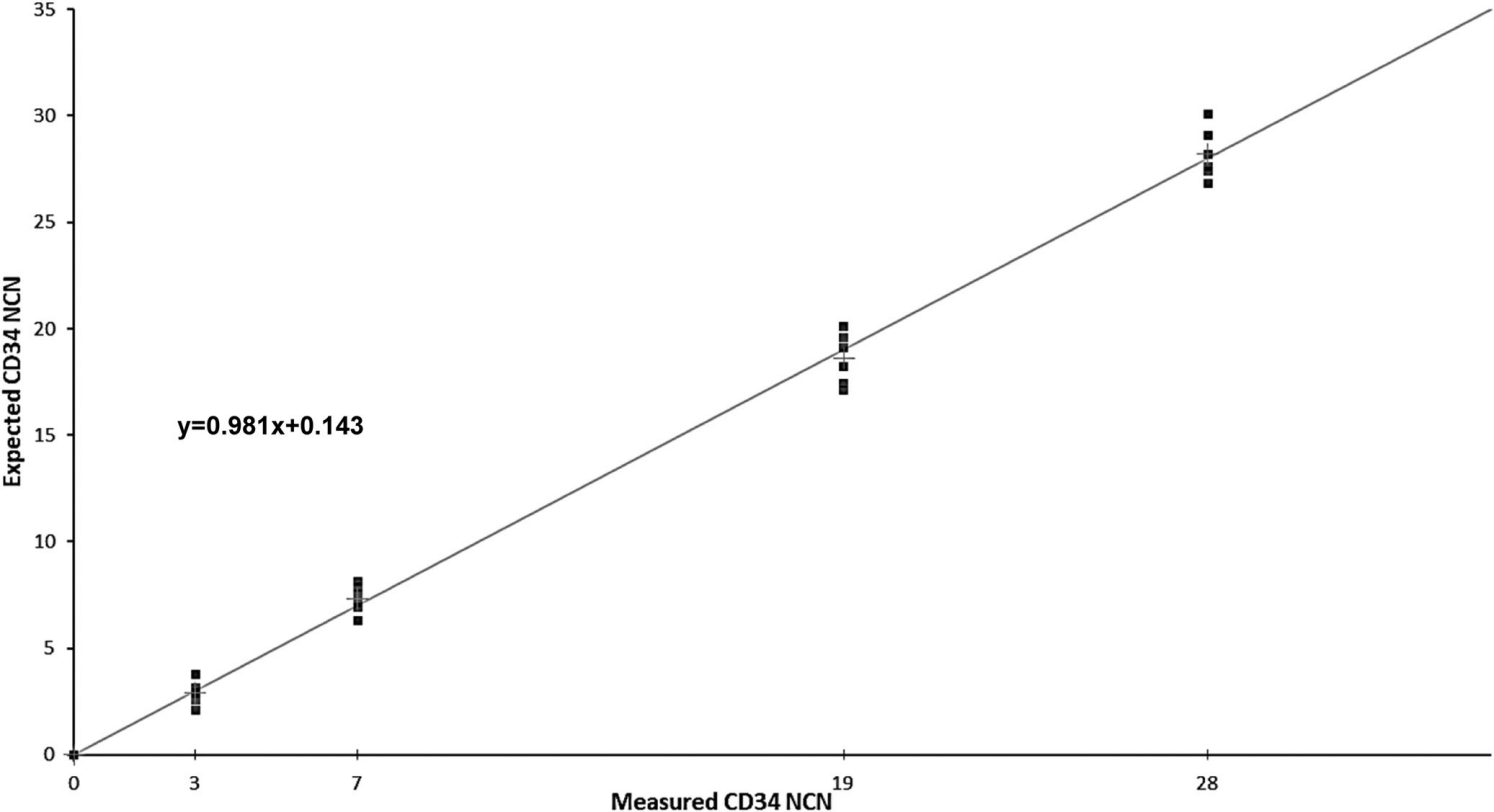
CD34 expression dynamic range. CD34 dynamic range was analyzed with a CD34 NCN (expected vs. measured) between 3 and 28. The maximal allowed difference between the expected and measured NCN was 10%.

The MU at a level above the LoQ (mean CD34 NCN 6.7 range 6–8.5) resulted in a CV of 10.5% and 12.5% for the intra and the interassay respectively. When the MU was tested at lower level (mean CD34 NCN 0.6 range 0.4–0.8) the CV was 13.4% and 23.3% for the intra and inter assay respectively.

### 3.3 CD34 expression assay diagnostic performance

The suggested TNC cell dose to be infused for HSCT lies between 2 and 4 × 10^8^ TNC per kilogram of recipient body weight. Therefore, a threshold value of 3 × 10^8^ TNC per kilogram of recipient body weight was used to split the CD34 NCN per kilogram of recipient body weight values into two cohorts, namely above or below the mentioned threshold value. By using this threshold value, 48 (68%) donors were above the threshold, while the remaining 23 (32%) donors showed values below the defined threshold. Afterwards, and to analyze the diagnostic performance of the assay, the true‐positive rate (sensitivity) and the false‐positive rate (1 − specificity) were plotted in a ROC space.

The ROC curve showed an area under the curve (AUC) of 0.96 (95% confidence interval 0.91–1.01, *p* < .005). The CD34 NCN per kilogram of recipient body weight threshold of 0.54 resulted in a sensitivity of 0.91, a specificity of 0.96, and a positive and negative predictive value of 0.97 and 0.91 respectively (Figure 3).

**FIGURE 3:**
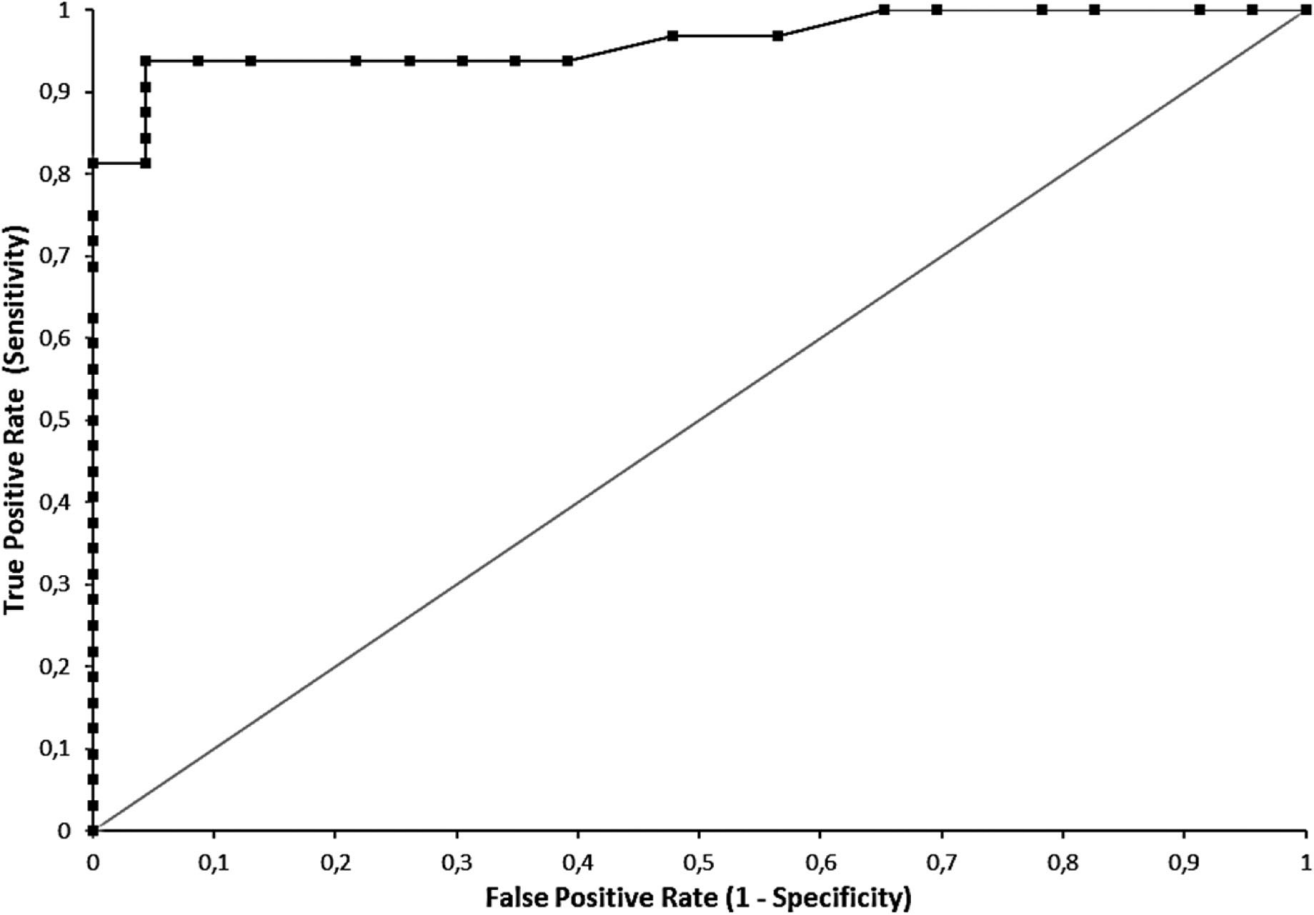
CD34 expression assay diagnostic performance. True positive rate (sensitivity) was plotted against the false positive rate (1 − specificity), resulting in a ROC curve. The diagonal line corresponded to random chance.

### 3.4 CD34 expression and BMH related characteristics

We next investigated the correlation between CD34 expression and several BMH parameters. A robust correlation between CD34 NCN per kilogram of recipient body weight and TNC per kilogram of recipient body weight was found (Spearman's *r*s 0.87, 95% CI: 0.78–0.92, *p* < .001) (Figure 4A). In addition, the correlation between CD34 NCN per kilogram of recipient body weight and CD34 cell count per kilogram of recipient body weight, assessed by flow cytometry, was also strong (Spearman's *r*s 0.91, 95% CI: 0.84–0.94, *p* < .001) (Figure 4B). A moderate although significant correlation between CD34 NCN per kilogram of recipient body weight and CD3 cell count per kilogram of recipient body weight was found (Spearman's *r*s 0.71, 95% CI: 0.54–0.82, *p* < .019) (Figure 4C). The BMH volume was negatively correlated with TNC count per kilogram of recipient body weight (Spearman's *r*s −0.703, 95% CI: −0.82 to 0.53, *p* < .02) (Figure 5A) and with CD34 NCN per kilogram of recipient body weight (Spearman's *r*s −0.732, 95% CI: −0.84 to 0.58, *p* < .006) (Figure 5B). No correlation between CD34 expression (expressed either as NCN or NCN per kilogram of recipient body weight) and donor age, sex or weight was observed.

**FIGURE 4:**
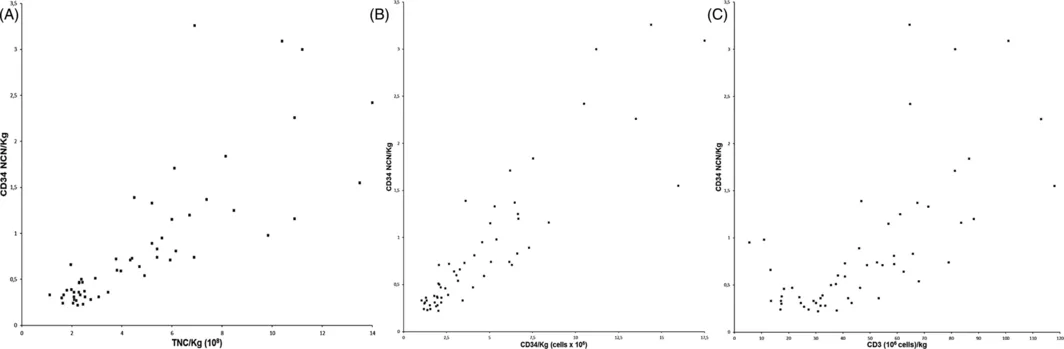
Correlation between CD34 expression assay and BMH parameters. (A) TNC/kg vs. CD34 NCN/kg. (B) CD34 expression detected by flow cytometry and CD34 NCN detected by dPCR. (C) CD3 expression vs. CD34 NCN. All findings are expressed by kilogram of recipient body weight.

**FIGURE 5:**
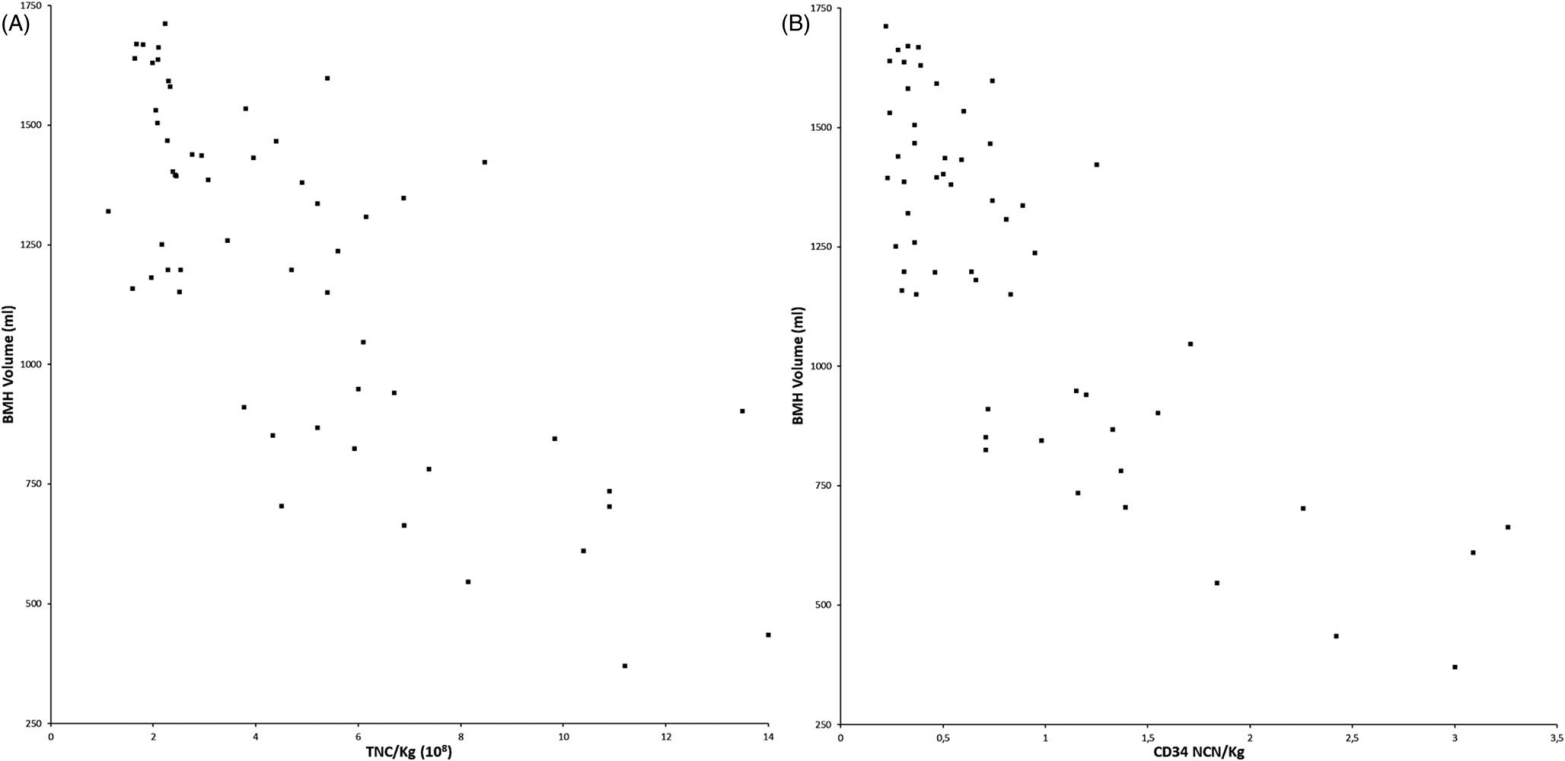
Correlation between BMH volume, TNC and CD34 expression. (A) BMH volume correlation with TNC count/kg. (B) BMH volume correlation with CD34 NCN/kg. Expressed by kilogram of recipient body weight.

## 4 DISCUSSION

Although PB is the main source of hematopoietic cells for HSCT, marrow is still the preferred graft source in certain clinical situations. Marrow HSCT has been associated with reduced incidence of chronic GvHD when compared with the use of PB as a graft source.^18^

The final number of collected TNC after the BMH procedure is predictive of successful engraftment, survival and non‐relapse mortality.^5^ During and after the BMH, the TNC counts are typically assessed with a hematology analyzer. However, the TNC is represented by a heterogeneous group of cells that includes different cell types such as lymphocytes, monocytes, neutrophils and non‐hematopoietic cells such as mesenchymal stem cells. From the collected TNC, only a minor fraction of cells has engraftment and long‐term repopulation capabilities. The CD34 cell marker, usually assessed by flow cytometry, is used to identify those cells with long‐term repopulation potential.^19^, ^20^ Besides flow cytometry, sensitive CD34 PCR‐based detection assays have been described although not widely implemented.^14^ In addition, in these reports the assay validation description is scarce and mainly focused on the analytical sensitivity. The assay described here has gone through a comprehensive validation process based on guidelines from different standardization entities.^15^, ^16^, ^17^ The mean CD34 expression found in the donor population is several times higher than the established LoQ and therefore the CD34 assay analytical sensitivity is adequate for the intended purpose. The linear regression analysis resulted in an assay dynamic range that covers most of the CD34 expression values in our donor cohort. However, some values were above the upper limit of the established dynamic range and it is uncertain if such values still have a linear distribution.

The assay MU showed moderate variability in the low‐level concentration range. Although a long‐term measurement using different reagent lots, instruments, and operators is needed for a more comprehensive precision evaluation.

To analyze the assay analytical performance, we decided to use a threshold value of 3 × 10^8^ TNC per kilogram of recipient body weight to split the CD34 NCN expression in the donor population because this threshold value lies between the suggested TNC dose to be infused for HSCT. The high sensitivity, positive predictive value, and diagnostic accuracy, represented by the AUC in the ROC curve, suggest that the CD34 NCN expression assay described here is a suitable accessory adjunct for TNC count assessment after BMH.

In line with a previous study, the CD34 expression assessed by real‐time PCR showed a strong correlation with the CD34 expression detected by flow cytometry.^21^ In addition, a similar degree of correlation between CD34 expression detected with our assay and the TNC count was observed.

Also as previously described, we observed a negative correlation between the TNC count, the CD34 expression and the collected marrow volume.^22^ This last finding suggests that increasing the collected marrow volume does not result in a TNC count increase and therefore reducing the BM volume and the procedure duration might be beneficial for the marrow donor.

Our study is not complete and several points need to be fulfilled. We have no access to the post‐transplantation data and therefore it was not possible to evaluate the relation between our CD34 expression assay performance and relevant transplant‐related parameters such as neutrophil and platelet regeneration time, infection rate, overall survival, and GvHD incidence among others. Further studies relating the TNC count together with the CD34 expression assay described here and post‐transplantation variables are needed to address the clinical usefulness of the CD34 expression assay. Recently, novel models based on deep learning algorithms have been developed to predict overall survival and transplant‐related complications.^23^, ^24^ One of these models includes the number of CD34 cells infused, and this parameter alone was able to predict survival with high accuracy.^25^

Taken together, our data suggest that the CD34 expression assay described here can be used as an additional tool for TNC count estimation after BMH, in particular when the TNC count performed at the harvesting facility does not match the TNC analysis at the transplantation center.

## AUTHOR CONTRIBUTIONS

MW designed and supervised the study. MW, AK, and MD established the methodology and analyzed the data. MS, DS, MP, and JR analyzed the flow cytometry data. RZ, CW, RW, JD, and JF were responsible for patient clinical care. C K‐S and SE were responsible for bone marrow harvest and donor data supervision.

## FUNDING INFORMATION

The authors have nothing to report.

## CONFLICT OF INTEREST STATEMENT

The authors have disclosed no conflicts of interest.

## Supporting information


**APPENDIX S1.** Supporting information.
**FIGURE S1:** Representative BMH label for the final collection bag.
**TABLE S1:** Primer/Probe sequence CD34 and ABL used in this study.
**FIGURE S2:** A temperature gradient (between 57 and 65°C) was performed and the optimal AT was 60°C (*R*s = 2.4).

## Data Availability

The data that support the findings of this study are available on request from the corresponding author. The data are not publicly available due to privacy or ethical restrictions.
