# Supplementary material for: Performance evaluation of a CD34 expression assay by digital PCR in bone marrow harvest from healthy donors
Source: Transfusion. 2026 Jun 1;66(11):2293–301. doi: 10.1111/trf.70267 (PMC13753073; doi:10.1111/trf.70267)
Supplement: Supplementary file 1 [file TRF-66-2293-s001.docx]

Supplementary material

Bone Marrow Harvest

The BMH procedure time was highly variable and this duration depends on several factors, such as anesthesia induction, skin desinfection and vital parameters documentation before the procedure among others. Of note, the BM volume to be collected did not have a significant influence in the whole BMH procedure time. Therefore, the BMH time frame for this study, was defined as the time point where the first aspiration was made up to the time of the last aspiration. The BMH procedure starts in the operating room with the donor identification done by the anesthesia team and the BMH operator. Afterwards, the donor in a prone position is sedated and intubated by the anesthesiologist according to institutional guidelines.

After careful skin sterilization the BMH site is covered with a fenestrated sheet, exposing the iliac spines and crests. The BM was harvested bilaterally from the iliac crest by two operators. With the needle inside the medullary space, the BM was aspirated with a 20 ml syringe. After aspiration, the BM is transferred to the collection bag by the nursing staff or a lab technologist. The collection bag was regularly and carefully massaged by hand during the procedure to ensure a complete mix of the BM and the anticoagulant solution.

After the last BM transfer, the BM contained in the collection bag was transferred through a filter series in a closed collection system to the final collection bag. This last bag was labeled with the appropriate information (donor ID, time of collection, BM volume, etc. Figure S1). From the final collection bag, BM samples were taken for cell count and sterility controls. The BM volume was calculated by weight using the BM specific gravity (1.058 g/ml).

Figure S1. Representative BMH label for the final collection bag

| **Lot:**  **SEC:**  **GRID:**  **G** | Gen.-Nr.: **PEI.G.04069.01.1**  Human allogeneic Hematopoietic Progenitor Cells (HPC) from bone marrow- frisch zurück U-FR-Med I |
| --- | --- |
| **Collection Date (dd/mm/yyyy):**  **2**  **Start: (EST)/ End: (EST)** |  |
| **Recipient-ID: xxx**  **Recipient name: xxx**  **DoB: xxx** | **For intended recipient only! Please also see Product Information!**  HUMAN CELLS FOR TRANSPLANTATION. DO NOT IRRADIATE! DELIVER IMMEDIATELY!  DO NOT USE LEUKOREDUCTION FILTERS! CHECK DONOR AND RECIPIENT IDENTITY! |
| **Volume: ml**  **ACD-A: ml**  **CD34^+^: x10^6^/kg**  **WBC: x10^8^/kg** | Storage and Transport at roomtemperature or 4°C to 8°C // Suspension for i.v. application |
|  | **Expiry Date (dd/mm/yyyy) / Time (EST):**  ***5 /*** |
|  | Unit 1 of 1 |
| ***Blood group:* xx Rh xx** | Date and Time of delivery: see Product Information |
| **Pharmaceutical Entrepreneur:**  **Universitätsklinikum Freiburg (U-FR), AöR**  **Hugstetter Str. 49, 79106 Freiburg**  **Manufacturer:**  **Uniklinik FR, Dept. Int. Med. I, Sekt. allogene Stammzelltranspl.**  **Hugstetter Str. 55, 79106 Freiburg**  **Tel.: 0049-761-270 33210 Fax: -36580353658035836365803658036580** |  |

Table S1. Primer/Probe sequence CD34 and ABL used in this study.

| Target gene | Primer sequence (5‘-3‘) | Probe sequence* (5‘-3‘) |
| --- | --- | --- |
| *CD34* Forward  Reverse | CCT GAT GAA TCG CCG CAG CTG GAG C  CCT GGC CTC CAC CGT TTT | CAC AGG AGA AAG GCT GGG CGA AGA CCC |
| *ABL* Forward  Reverse | TGGAGATAACACTCTAAGCATAACTAAAGGT  GATGTAGTTGCTTGGGACCCA | CCA TTT TTG GTT TGG GCT TCA CAC CAT T |

**CD34* and *ABL* Probe were labelled with FAM and HEX respectively

Annealing temperature: resolution factor (Rs)

As suggested by the European commission joint research center for digital PCR assays, the resolution factor (Rs) was used to estimate the optimal annealing temperature (AT) for the complete separation of the positive and negative fluorescence clusters. The Rs is represented by the following equation:

𝑅𝑠 = 2 x (Fa 𝑝 – Fa 𝑛) /𝑤𝑝 + 𝑤𝑛

Fa corrrespond to the fluorescence amplitude and w to the peak width. p and n indicates positive and negative fluorescence respectively.

Figure S2. A temperature gradient (between 57 and 65 ⁰C) was performed and the optimal AT was 60 ⁰C (Rs= 2.4)
